# Specimen rejections among referred specimens through referral network to the Amhara Public Health Institute for laboratory testing, Bahir Dar, Ethiopia

**DOI:** 10.1186/s13104-018-3891-7

**Published:** 2018-11-01

**Authors:** Melashu Balew Shiferaw, Gizachew Yismaw, Hailu Getachew

**Affiliations:** Amhara Public Health Institute, P.O.Box 447, Bahir Dar, Amhara, Ethiopia

**Keywords:** Rejection, Specimen, Referral network, Laboratory, Ethiopia

## Abstract

**Objective:**

The aim of this study was to assess the magnitude, trend and reasons of rejection among referred specimens through referral network to the Amhara Public Health Institute (APHI) for laboratory testing.

**Results:**

A total of 42,923 specimens were received at APHI reference laboratories. Of which, 221 (0.5%) specimens were rejected. CD4, HIV viral load, genexpert and EID specimens’ rejection rates were 0.7%, 0.6%, 0.3% and 0.2%, respectively. CD4 specimens were rejected due to wrong package (84.2%) and presence of clots (15.8%). Un-centrifuge (46.9%), hemolysis (19.8%) and use of wrong tube (17.7%) were the main rejection reasons for HIV viral load specimens. Although viral load specimen rejection was improved from 1.8 to 0% up to February/2018, the problem was reoccurred and continued to the end of May (1.3%) and June (0.3%) 2018. Moreover, CD4 specimen rejection (4.3%) was out of the established target in May, and exposed infant diagnosis (EID) specimen rejection became increased since March 2018. Hence, appropriate corrective and preventive actions and close follow up could reduce the problem of specimen referral network.

## Introduction

About 70% of the errors in the laboratory occur during pre-analytical phase of the laboratory processes [1]. Specimen collection is one of the pre-analytical processes that ensure to provide accurate, reliable and timely results to patients. However, improper collection of samples could delay patient results due to unnecessary specimen re-draws and elongated corrective and preventive action activities. This could dissatisfy customers in addition to time and resource wastage in the laboratory [2].

Therefore, quality of the laboratory results is good only if the quality of specimen collection and transportation is appropriate. It is clear that poor laboratory results could influences the diagnosis and therapeutic decisions mainly impacting implementation of proper patient managements and its outcomes. So that the laboratory must work on the pre-set standards to ensure received samples maintain its integrity to generate reliable and timely patient results [2–4].

Frequently, samples are collected outside the laboratory, and transported for testing. In this case, transport must be managed carefully in order to maintain sample integrity, temperature, preservation needs, special transport containers, and time limitations. Personnel who package or transport the specimen should be trained about the proper procedures, both for safety and for good maintenance of samples [5–7].

According to the International Organization for Standardization (ISO), clinical laboratories should develop criteria for acceptance or rejection of samples. Problems with patient or sample identification, sample instability due to delay in transport or inappropriate container(s) and insufficient sample volume are some of the examples of rejection criteria. However, when the sample is clinically critical or irreplaceable, the laboratory chooses to process the sample, and the final report should indicate the nature of the problem and, where applicable, that caution is required when interpreting the result [8].

When samples are rejected, it is important to inform authorized person that the sample is unsuitable for testing, and request another sample to be collected again. However, the laboratory should retain rejected sample pending a final decision regarding disposition. As a continuous quality improvement, the management should regularly review the number of rejected samples and reasons for rejections, conduct training on sample collection, and revise written procedures for sample management as needed [4].

In Ethiopia, laboratory testing is through sample referral to more advanced reference laboratories using the established referral networking system [9]. A study conducted in Gondar University hospital showed that specimen rejection contributed 3.8% of the total pre-analytical errors for clinical chemistry tests [10]. However, detailed information of different specimens such as for viral load, Tuberculosis (TB) genexpert, EID and CD4 tests linked through specimen referral network is limited. Therefore, the aim of this study is to assess the status of specimen rejection among received specimens through sample referral networking system in APHI reference laboratories.

## Main text

### Methods

A retrospective study was carried out from August 08-30, 2018. Documents of patient specimens sent from referring sites to the Amhara Public Health Institute for laboratory investigation were reviewed for its quality retrospectively. Under Amhara public health institute health facilities were linked for referral testings. There were 10 treatment initiation centers for TB culture diagnosis, 107 health facilities for viral load and EID testing and 16 health facilities linked for CD4 and chemistry tests. Tracking records of received specimens from July 1, 2017 to June 30, 2018 at the APHI were included and reviewed. Records with incomplete information were excluded from the study.

Data extraction tool was prepared by the authors and used to capture information regarding specimen rejections and specimen management systems established at the APHI reference laboratories. Monthly quality indicator reports, which included specimen rejection and workload statistics, were also used as data base. Each month, APHI reference laboratories (molecular, measles/rubella, TB, Immunohematology, Clinical chemistry and parasitology laboratories) report monthly quality indicators to the quality coordination office as a continuous quality improvement.

Data clerks collected the data after brief orientation on the extraction tool, type of data to be included and the source of data to be reviewed. In APHI, all the specimens received from referring sites and/or collected in the site were tracked and evaluated by the central reception, and labeled using barcode. The central reception used Polytech Laboratory Information System (Comp Pro Med, Inc., USA) to transfer requisition and get patient report from the reference laboratories. Then, the specimens were delivered to each laboratory and evidenced with recording in the internal delivery format.

Data were extracted from the excel data base prepared for tracking of patient specimens, from polytech LIS and from the monthly quality indicators report. Then, the data were transferred to SPSS version 20.0 (IBM Corporation, USA) for analysis after cleaning. Descriptive statistics like frequencies and proportions were presented to show the problems related with specimen rejections and management practices.

### Results

In this study, a total of 42,923 specimens were submitted at the central reception of the APHI reference laboratories. Majority of the specimens, 34,101 (79.5%), were for viral load testing to monitor effectiveness of HIV antiretroviral drugs. Line probe assay (LPA), TB drug resistance testing, was the least numbers of specimens with 55 (0.2%) specimens. A total of 354 specimens were submitted for clinical chemistry tests such as ALT (alanine aminotransferase), AST (aspartate aminotransferase), creatinine, and alkaline phosphatase in the institute. TB culture specimens accounted about 3% of the total specimens received. Specimens for exposed infant diagnosis of HIV, CD4, TB genexpert and measles/rubella accounted 4.8%, 6.4% and 1.1% of the total submitted specimens, respectively (Table 1).

**Table 1:** Referral specimens submitted at the central reception of the APHI for laboratory investigation from 01 July 2017 to 30 June 2018

Tests	Number of specimen submitted	%
HIV 1 viral load	34,101	79.5
EID	2056	4.8
CD4	2761	6.4
Clinical chemistry	354	0.8
TB culture	1171	2.7
TB genexpert	1938	4.5
LPA	55	0.2
Measles and Rubella	487	1.1
Total	42,923	100%

Among the total submitted specimens, 221 (0.5%) were rejected due to poor quality of the specimens that did not fulfill the necessary requirements to be tested. The rejection rates varied among specific specimens. Routine viral load, EID, CD4, and TB genexpert tests were among the rejected samples. Majority of the rejected specimens were HIV viral load (192 [0.6%]) and CD4 (19 [0.7%]). CD4 specimens were rejected due to wrong package (84.2%) and presence of clots (15.8%).Un-centrifuge specimens (46.9%), hemolyzed specimens (19.8%) and use of wrong tube (17.7%) were the main rejection reasons for viral load specimens. A total of five EID specimens (0.2%) were rejected due to labeling problem and poor packaging (mixed together with icepack) during transportation of the DBS. Similarly, five genexpert specimens were rejected due to use of wrong collection tube (2/5) and labeling problems (2/5). Interestingly, there was no rejection for clinical chemistry, measles, LPA and TB culture (Table 2).

**Table 2:** Number of rejected specimens and reasons in APHI reference laboratories from 01 July 2017 to 30 June 2018

Tests	Number of rejected specimens	Specimen rejection reasons								
		Long storage/> 5 days	Packed with/without ice pa	Un-centrifuge	Hemolyzed	Insufficient volume	Clotted	Wrong tube/leaked	Labeling problem	Bloody specimen
HIV 1 viral load	192	14	12	90	38	1	3	34	0	0
EID	5	0	2	0	0	1	0	0	2	0
CD4	19		16	0	0	0	3	0	0	0
Clinical chemistry	0	0	0	0	0	0	0	0	0	0
TB culture	0	0	0	0	0	0	0	0	0	0
genexpert	5	0	0	0	0	0	0	2	2	1
LPA	0	0	0	0	0	0	0	0	0	0
Measles and Rubella	0	0	0	0	0	0	0	0	0	0
Total	221	14	30	90	38	2	6	36	4	1

Specimens for HIV viral load testing were rejected. The rejections were higher in the first 5 months since July to November 2017 with rejection rates ranging from 1.0 to 1.8%. Then, it was improved in the next 3 months (December 2017 to February 2018) with zero specimen rejections. However, the rejection reoccurred in March (0.2%), May (1.3%) and June (0.3%). Surprisingly, at least one EID specimens was rejected per month from March to June. CD4 specimens were rejected in December, May and June with rejection rates 1.0%, 4.3% and 0.4%, respectively (Table 3).

**Table 3:** Trends of rejected specimens in APHI reference laboratories from 01 July 2017 to 30 June 2018

Specimens	2017						2018					
	Jul	Aug	Sept	Oct	Nov	Dec	Jan	Feb	Mar	Apr	May	June
HIV 1 viral load												
Total received	2076	823	2041	3393	2425	3709	3725	2497	4192	4057	2637	2526
Rejected	26	15	31	35	33	0	0	0	9	0	35	8
%	1.3	1.8	1.5	1.0	1.4	0.0	0.0	0.0	0.2	0.0	1.3	0.3
EID												
Total received	0	439	165	57	45	319	403	135	217	74	165	101
Rejected	0	0	0	0	0	0	0	0	1	1	1	2
%	–	0.0	0.0	0.0	0.0	0.0	0.0	0.0	0.5	1.4	0.6	2.0
CD4												
Total received	0	0	0	154	274	294	393	401	390	278	348	229
Rejected	0	0	0	0	0	3	0	0	0	0	15	1
%	–	–	–	0.0	0.0	1.0	0.0	0.0	0.0	0.0	4.3	0.4
TB genexpert												
Total received	78	75	114	168	170	320	190	197	186	159	167	114
Rejected	0	1	0	0	0	0	0	2	2	0	0	0
%	0.0	1.3	0.0	0.0	0.0	0.0	0.0	1.0	1.1	0.0	0.0	0.0

### Discussion

This study investigates the magnitude of rejection among received specimens through sample referral network. Genexpert for TB drug resistance testing, HIV viral load, CD4 and EID specimens were among the rejected specimen types. We also evaluated the rejection reasons and trends of rejection whether the problem was improved through time or continued as it was.

Data on rejected samples due to various types of pre-analytical errors is one of the pre-analytical quality indicators [8]. The APHI established specimen rejection rate of 2% or below as a monthly quality indicator. In this study, a total of 221 specimens did not fulfill the necessary requirement that makes a rejection rate of 0.5%. Even though the institute achieved the established target, the magnitude of this rejection is high that needs preventive actions since improper collection of specimens could cause delay in reporting, incorrect diagnosis or treatment, and death [2]. The finding of this study is also comparable with a study conducted in Turkey that showed total rejection rate of 0.65% [11]. Similarly, the prevalence of pre-analytical problems was documented from 0.2 to 0.75% [12]. Appropriate training, communication and follow up on specimen collection could reduce the problem as a preventive action [13].

Referral testing requires proper packaging and shipping of patient specimens to preserve their integrity and suitability and to protect all persons involved in their transportation. In the present study, 0.7% of the CD4 specimens were rejected. Of which, 84.2% of the rejections were due to wrong packages. The packaging and transportation must comply with the transportation of dangerous goods regulations. So that, CD4 specimens should be packed and transported at room temperature to maintain the integrity of the specimen [14].

Specimen integrity is the cornerstone of a quality viral load test result. To protect specimen integrity, they must be properly collected in the correct type of tube, stored at the correct temperature, properly processed and within the proper timeframe, transported in the right temperature and packaging. EDTA derived plasma requires centrifugation [15]. In this study, 0.6% (192/34,101) of the viral load specimens was rejected. Un-centrifuge (46.9%) and hemolysis (19.8%), and use of wrong tube (17.7%) were the main rejection reasons for viral load specimens. Reasons for un-centrifuged and use of wrong tube needs further investigations why the health facilities did not submit plasma specimen. There may be a shortage of centrifuge equipment and/or knowledge gap due to inappropriate training. Moreover, hemolysis could influence test results by falsely elevating the analytes [16]. Vigorous mixing of the specimen, pneumatic tube transport of the specimens, or forcing of blood through a large-bore needle of a syringe may cause the red blood cells to rupture, resulting in hemolysis [17].

Although viral load specimen rejection was improved from 1.8 to 0% up to February 2018, the problem was reoccurred and continued to the end of May (1.3%) and June (0. 3%). Moreover, EID specimen rejection became increased since March 2018. This needs strict preventive and corrective actions and, close follow up to improve the laboratory service more.

## Limitations

This study used secondary data from record review of the specimen tracking and monthly quality indicators report in APHI. If data were not recorded, this could underestimate the magnitude of specimen rejection. In addition, some important variables like training status of sample transporters such as couriers and drivers on sample management and safety was missed.

## Abbreviations

APHI: Amhara Public Health Institute
EID: exposed infant diagnosis
LPA: line probe assay
TB: tuberculosis
